# 粪菌移植治疗异基因造血干细胞移植后糖皮质激素耐药胃肠道急性移植物抗宿主病19例临床研究

**DOI:** 10.3760/cma.j.issn.0253-2727.2023.05.008

**Published:** 2023-05

**Authors:** 雨雨 郑, 萧天 杨, 国强 林, 梅茹 卞, 叶俊 司, 兴霞 张, 彦明 张, 德沛 吴

**Affiliations:** 1 徐州医科大学附属淮安医院，淮安 223002 Department of Hematology, Huai'an Hospital Affiliated to Xuzhou Medical Universitity, Huai'an 223002, China; 2 苏州大学附属第一医院血液科，江苏省血液研究所，国家血液系统疾病临床医学研究中心，苏州 215006 Department of Hematology, the First Affiliated Hospital of Soochow University, Jiangsu Institute of Hematology, National Clinical Research Center for Hematologic Disease, Suzhou 215006, China

**Keywords:** 异基因造血干细胞移植, 急性移植物抗宿主病, 肠道菌群, 粪菌移植, 淋巴细胞亚群, 炎症因子, Allogeneic hematopoietic stem cell transplantation, Acute graft-versus-host disease, Intestinal microbiota, Fecal microbiota transplantation, Lymphocyte subpopulation, Inflammatory cytokines

## Abstract

**目的:**

探讨粪菌移植（FMT）治疗糖皮质激素耐药胃肠道急性移植物抗宿主病（GI-aGVHD）的临床疗效。

**方法:**

纳入2017年3月至2022年3月期间在徐州医科大学附属淮安医院行异基因造血干细胞移植（allo-HSCT）后发生糖皮质激素耐药GI-aGVHD的29例血液病患者，其中19例行FMT治疗（FMT组），10例未接受FMT治疗（对照组）。观察疗效及安全性，分析FMT治疗前后肠道菌群丰度、淋巴细胞亚群比例、外周血炎症因子及GVHD生物标志物的变化。

**结果:**

①13例患者（68.4％）在FMT后临床症状达到完全缓解，对照组2例患者（20.0％）达到完全缓解，差异有统计学意义（*P*<0.05）。FMT后肠道微生物群多样性增加，并逐渐恢复至正常水平，未发生FMT相关感染。②与对照组比较，FMT组治疗后CD3^+^、CD8^+^细胞占比降低，CD4^+^、调节性T细胞（Treg）及CD4^+^/CD8^+^细胞比值升高（*P*值均<0.05），IL-6浓度低于对照组［4.15（1.91～5.71）ng/L对6.82（2.40～8.91）ng/L，*P*＝0.040］，IL-10浓度高于对照组［12.11（5.69～20.36）ng/L对7.51（4.10～9.58）ng/L，*P*＝0.024］。胰岛衍生蛋白3α（REG3α）在发生GI-aGVHD时明显升高，FMT组治疗后REG3α水平低于对照组［30.70（10.50～105.00）µg/L对74.35（33.50～139.50）µg/L，*P*＝0.021］。

**结论:**

FMT是糖皮质激素耐药GI-aGVHD的有效、安全治疗方法。FMT可促进肠道菌群多样性的恢复、调节炎症因子和上调Treg细胞表达。

## Abstract (en)

**Objective:**

To investigate the clinical efficacy of fecal microbiota transplantation（FMT）for treating steroid-refractory gastrointestinal acute graft-versus-host disease（GI-aGVHD）.

**Methods:**

This analysis included 29 patients with hematology who developed steroid-refractory GI-aGVHD after allogeneic hematopoietic stem cell transplantation（allo-HSCT）in Huaian Hospital Affiliated to Xuzhou Medical University from March 2017 to March 2022. Among them, 19 patients underwent FMT treatment（the FMT group）and 10 patients did not（the control group）. The efficacy and safety of FMT were assessed, as well as the changes in intestinal microbiota abundance, lymphocyte subpopulation ratio, peripheral blood inflammatory cytokines, and GVHD biomarkers before and after FMT treatment.

**Results:**

① Complete remission of clinical symptoms after FMT was achieved by 13（68.4％）patients and 2（20.0％）controls, with a statistically significant difference（*P*<0.05）. Intestinal microbiota diversity increased and gradually recovered to normal levels after FMT and FMT-related infections did not occur. ②The proportion of CD3^+^ and CD8^+^ cells in the FMT group after treatment decreased compared with the control group, and the ratio of CD4^+^, regulatory T cells（Treg）, and CD4^+^/CD8^+^ cells increased（all *P*<0.05）. The interleukin（IL）-6 concentration in the FMT group was lower than that in the control group［4.15（1.91–5.71）ng/L *vs* 6.82（2.40–8.91）ng/L, *P*＝0.040］, and the IL-10 concentration in the FMT group was higher than that in the control group［12.11（5.69–20.36）ng/L *vs* 7.51（4.10–9.58）ng/L, *P*＝0.024］. Islet-derived protein 3α（REG3α）was significantly increased in patients with GI-aGVHD, and the REG3α level in the FMT group was lower than that in the control group after treatment［30.70（10.50–105.00）µg/L *vs* 74.35（33.50–139.50）µg/L, *P*＝0.021］.

**Conclusion:**

FMT is a safe and effective method for the treatment of steroid-refractory GI-aGVHD by restoring intestinal microbiota diversity, regulating inflammatory cytokines, and upregulating Treg cells.

胃肠道急性移植物抗宿主病（GI-aGVHD）是异基因造血干细胞移植（allo-HSCT）的常见并发症，Ⅲ/Ⅳ级GI-aGVHD严重影响患者的预后及生活质量[1]。GI-aGVHD主要表现为水样腹泻、腹痛、便血和肠梗阻，导致移植相关早期死亡率增高[2]。目前，GI-aGVHD的一线治疗主要是糖皮质激素，但近半数患者耐药[3]，二线治疗可选择他克莫司、霉酚酸酯（MMF）、CD25单抗、肿瘤坏死因子-α（TNF-α）单抗、抗胸腺细胞球蛋白（ATG）、芦可替尼、间充质干细胞（MSC）等，但目前尚无标准的二线治疗方案。

以往研究显示肠道微生物群在allo-HSCT后aGVHD的发生和发展中起重要作用，微生物多样性的丧失可促进aGVHD的发展[4]。因此，调节肠道菌群稳态和维持有益菌的优势是治疗aGVHD的可行方法。粪菌移植（FMT）已被证明可恢复肠道菌群的多样性[5]，一些小样本研究也显示FMT治疗难治性aGVHD取得良好疗效[6]–[8]。我们采用FMT治疗19例allo-HSCT后糖皮质激素耐药GI-aGVHD患者，观察其疗效、安全性并分析肠道菌群丰度及对免疫稳态的影响。

## 病例与方法

一、病例

本研究为回顾性分析，纳入2017年3月至2022年3月在我院行allo-HSCT后发生糖皮质激素耐药的29例GI-aGVHD患者，其中19例行FMT治疗（FMT组），另10例未接受FMT治疗（对照组）。依据GVHD国际联盟（MAGIC）标准[10]进行诊断及分级，满足以下任一条即可诊断糖皮质激素耐药GI-aGVHD：①糖皮质激素治疗3 d疾病进展、7 d无改善或14 d未缓解；②糖皮质激素减量期间治疗失败。所有患者均接受标准皮质糖皮质激素一线治疗和至少一种二线免疫抑制治疗。所有患者均知情同意并签署知情同意书。

二、FMT治疗方案及菌群多样性分析

FMT组19例患者中14例采用液态粪菌制剂（南京粪菌银行产品），5例采用粪菌胶囊（上海宝藤生物公司产品）。FMT前12 h停止使用抗生素，解冻复温的液态粪菌通过鼻十二指肠管注入1～3次，每治疗量3～8单位；粪菌胶囊每治疗量6单位，在1～4 d内分次口服。收集患者治疗前后粪便样本并储存于−80 °C条件下，委托上海宝藤生物公司进行16S rRNA基因测序，用属水平显示粪便细菌的组成，利用香农-维纳多样性指数来描述菌群的多样性。

三、疗效和安全性评价

根据FMT后14 d和28 d内腹痛、腹泻等症状的严重程度评估FMT疗效。

腹痛评分：轻度疼痛（1分），中度疼痛（2分），无干预的严重疼痛（3分）和严重疼痛（4分）。

疗效评价标准[11]：①完全缓解（CR）：腹泻、腹痛和（或）出血消失，或大便量在3 d内平均减少≥500 ml；②部分缓解（PR）：血便未完全消失但较最严重时减量超过50％，或日均便量>500 ml但较最严重时减量超过500 ml；③疾病进展（PD）：出现新发症状或原有症状加重或便量增量超过50％；④不符合以上标准者定义为疾病稳定（SD）。

根据FMT期间的不良事件和随访情况评估安全性。

四、淋巴细胞亚群检测

采用流式细胞术检测基线、发病时及治疗后外周血淋巴细胞亚群比例。

五、炎症因子及aGVHD生物标志物检测

采用全自动化学发光免疫分析仪（双抗体夹心酶联免疫分析法）检测患者基线、发病时及治疗后血清炎症因子及胰岛衍生蛋白3α（REG3α）的浓度，按说明书操作。

六、随访

随访截至2022年6月。总生存（OS）期定义为从GI-aGHVD诊断至患者死亡或随访截止的时间。无进展生存（PFS）期定义为患者接受治疗至任何原因所致复发、死亡或随访截止的时间。

七、统计学处理

采用SPSS 26.0软件进行统计学分析。计量资料用“中位数（范围）”表示，组间比较采用Mann-Whitney *U*检验。计数资料用“例（％）”表示，组间比较采用Fisher精确检验。生存曲线用Kaplan-Meier法绘制，组间生存期比较采用Log-rank检验。所用检验均为双侧，*P*<0.05为差异有统计学意义。

## 结果

一、病例资料

两组患者间性别、年龄、疾病类型、移植前疾病状态等基线特征差异无统计学意义（*P*>0.05）（表1）。所有患者均接受标准糖皮质激素一线治疗和至少一种二线免疫抑制治疗（环孢素A、他克莫司、CD25单抗、布地奈德等）。

**表1:** 29例糖皮质激素耐药胃肠道急性移植物抗宿主病（GI-aGVHD）患者临床资料

基本特征	FMT组（19例）	对照组（10例）	*P*值
年龄［岁，*M*（范围）］	30（13~60）	41（17~61）	0.456
性别［例（%）］			0.270
男	12（63.2）	4（40.0）	
女	7（36.8）	6（60.0）	
疾病类型［例（%）］			0.089
AML	10（52.6）	2（20.0）	
ALL	2（10.5）	3（30.0）	
CML	1（5.3）	0（0.0）	
MDS	3（15.8）	5（50.0）	
SAA	3（15.8）	0（0.0）	
移植前疾病状态［例（%）］			0.699
完全缓解	12（63.2）	7（70.0）	
非完全缓解	4（21.1）	3（30.0）	
非恶性肿瘤	3（15.8）	0（0.0）	
供者类型［例（%）］			1.000
同胞全相合供者	3（15.8）	2（20.0）	
单倍体供者	15（78.9）	8（80.0）	
无关供者	1（5.3）	0（0.0）	
干细胞来源［例（%）］			0.657
BM+PBSC	7（36.8）	2（20.0）	
PBSC	7（36.8）	4（40.0）	
PBSC+UCB	4（21.1）	4（40.0）	
BM	1（5.3）	0（0.0）	
预处理方案［例（%）］			0.397
改良BU/CY+ATG	12（63.2）	5（50.0）	
改良BU/CY	4（21.1）	1（10.0）	
TBI/CY	0（0.0）	1（10.0）	
DAC+改良BU/CY	2（10.5）	2（20.0）	
AZA+改良BU/CY	0（0.0）	1（10.0）	
Cla+改良BU/CY	1（5.3）	0（0.0）	
预处理方案含ATG［例（%）］			1.000
是	17（89.5）	9（90.0）	
否	2（10.5）	1（10.0）	
急性GVHD分级			1.000
Ⅱ级	2（10.5）	1（10.0）	
Ⅲ/Ⅳ级	17（89.5）	9（90.0）	
GI-aGVHD发病时间［d，*M*（范围）］	61（22~133）	45（13~150）	0.332
中位随访时间［月，*M*（范围）］	11（3~42）	7.5（2~57）	0.213

**注** FMT：粪菌移植；AML：急性髓系白血病；ALL：急性淋巴细胞白血病；CML：慢性髓性白血病；MDS：骨髓增生异常综合征；SAA：重型再生障碍性贫血；BM：骨髓；PBSC：外周血干细胞；UCB：脐带血；BU：白消安；CY：环磷酰胺；TBI：全身放射治疗；ATG：抗胸腺细胞球蛋白；DAC：地西他滨；AZA：阿扎胞苷；Cla：克拉屈滨

二、FMT疗效评估及安全性

患者疗效评估见表2。治疗后14 d，FMT组10例（52.6％）获得CR，6例获得PR，16例（89.5％）有效（CR+PR）；对照组1例（10.0％）获得CR，3例获得PR，两组有效率、CR率差异有统计学意义（*P*＝0.004，*P*＝0.032）。治疗后28 d，FMT组13例（68.4％）获得CR，对照组2例（20.0％）获得CR，差异有统计学意义（*P*＝0.021）。19例中1例CR患者出现恶心、呕吐症状，对症治疗后缓解，3例患者出现GVHD进展，表现为恶心、呕吐及血便。29例患者GVHD治疗过程中有3例发生菌血症或多重耐药菌感染，其中1例发生在对照组，1例发生在FMT治疗前，1例发生在FMT治疗1个月后未缓解患者。未出现FMT相关感染。

**表2:** 29例allo-HSCT后糖皮质激素耐药肠道急性移植物抗宿主病（GI-aGVHD）患者疗效评估

疗效指标	FMT组（19例）	对照组（10例）	*P*值
0 d			
日腹泻量［ml，*M*（范围）］	843（430~2200）	710（380~1400）	0.028
日腹泻频率［次，*M*（范围）］	12（5~23）	7（3~20）	0.069
腹痛评分［分，*M*（范围）］	4（3~4）	4（3~4）	0.378
14 d			
日腹泻量［ml，*M*（范围）］	470（90~1500）	645（200~900）	0.022
日腹泻频率［次，*M*（范围）］	4（2~15）	7（3~9）	0.038
腹痛评分［分，*M*（范围）］	2（0~4）	2（1~4）	0.061
CR+PR［例（%）］	16（84.2）	4（40.0）	0.032
CR［例（%）］	10（52.6）	1（10.0）	0.004
SD［例（%）］	2（10.5）	4（40.0）	0.143
PD［例（%）］	1（5.3）	2（20.0）	1.000
28 d			
日腹泻量［ml，*M*（范围）］	250（120~889）	330（210~1400）	0.077
日腹泻频率［次，*M*（范围）］	2（1~10）	3（2~7）	0.062
腹痛评分［分，*M*（范围）］	1（0~4）	2（0~4）	0.162
CR+PR［例（%）］	15（78.9）	6（60.0）	0.390
CR［例（%）］	13（68.4）	2（20.0）	0.021
SD［例（%）］	1（5.3）	1（10.0）	1.000
PD［例（%）］	3（15.8）	3（30.0）	0.633

**注** CR：完全缓解；PR：部分缓解；SD：疾病稳定；PD：疾病进展

三、GI-aGVHD发生时及FMT治疗后肠道微生物群特征

在GI-aGVHD发生时患者肠道微生物多样性明显降低，FMT组和对照组间差异无统计学意义（*P*＝0.377，图1A）。FMT组FMT治疗后患者肠道菌群多样性明显高于治疗前及非FMT组（*P*＝0.001，*P*＝0.012，图1A）。与对照组相比，FMT治疗组肠道拟杆菌属、梭菌属、双歧杆菌属的丰度显著升高（*P*<0.05），肠球菌属、阿克曼菌属的丰度显著下降（*P*<0.05）（图1B）。FMT治疗后有2例患者肠道菌群未见明显改善。

**图1:**
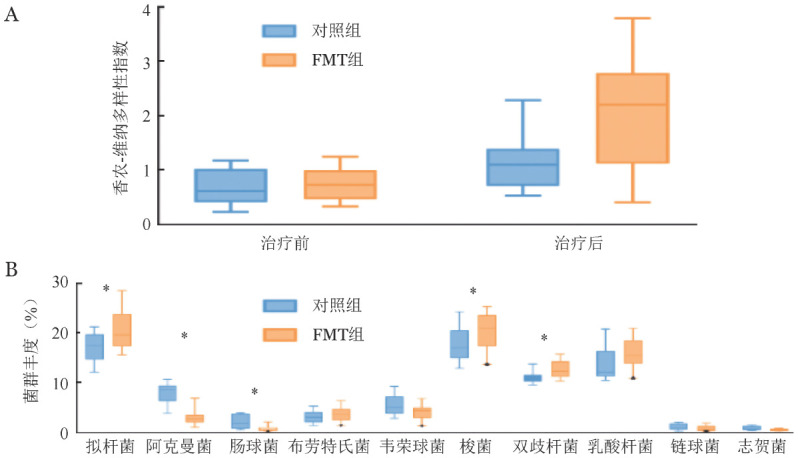
糖皮质激素耐药肠道急性移植物抗宿主病（GI-aGVHD）患者的粪便微生物群分析（A FMT组、对照组治疗前后香农-维纳多样性指数；B 前10个丰度最高属的相对丰度箱式图，最小平均丰度为>0.01。*P<0.05）

四、FMT治疗对淋巴细胞亚群影响

两组间淋巴细胞亚群在治疗前差异无统计学意义（*P*>0.05）。治疗后，FMT组CD3^+^、CD8^+^低于对照组，差异有统计学意义（*P*＝0.036，*P*＝0.040）。FMT组CD4^+^、Treg、CD4^+^/CD8^+^细胞比值高于对照组，差异有统计学意义（*P*＝0.023、*P*＝0.036、*P*＝0.005）（表3）。

**表3:** allo-HSCT后糖皮质激素耐药GI-aGVHD患者FMT治疗后外周血淋巴细胞亚群检测结果［*M*（范围）］

检测项目	基线	GVHD发病时	FMT治疗后
CD3^+^细胞（%）			
对照组	66.51（48.21~75.45）	73.72（49.71~94.47）	72.77（58.92~77.65）
FMT组	70.14（43.20~84.71）	80.15（47.26~92.60）	62.42（31.50~75.24）^a^
CD4^+^细胞（%）			
对照组	9.94（7.57~36.64）	7.65（5.15~9.15）	9.32（6.75~23.35）
FMT组	12.59（8.02~41.24）	9.04（5.04~20.64）	15.48（8.06~29.17）^a^
CD8^+^细胞（%）			
对照组	50.97（41.64~59.95）	63.47（46.15~81.30）	62.95（46.87~70.40）
FMT组	46.07（18.94~81.80）	57.68（44.78~87.12）	46.28（25.97~69.10）^a^
CD4^+^/CD8^+^细胞比值			
对照组	0.19（0.16~0.76）	0.12（0.09~0.16）	0.15（0.13~0.48）
FMT组	0.30（0.10~1.21）	0.16（0.06~0.45）	0.28（0.12~0.76）^a^
Treg（%）			
对照组	3.29（1.43~13.67）	0.62（0.00~3.21）	1.71（0.96~3.33）
FMT组	8.24（0.56~26.67）	0.11（0.00~5.50）	8.86（0.87~22.22）^a^
B细胞（%）			
对照组	0.89（0.22~1.78）	0.19（0.00~0.84）	0.68（0.12~1.37）
FMT组	0.90（0.03~4.07）	0.12（0.00~1.12）	0.56（0.04~4.30）
NK细胞（%）			
对照组	17.56（8.83~41.72）	9.43（3.70~28.94）	18.52（9.14~46.37）
FMT组	21.98（5.34~34.58）	13.46（3.52~29.80）	26.26（7.92~57.91）
NKT细胞（%）			
对照组	7.72（2.83~15.62）	1.76（0.53~9.48）	8.63（1.20~12.64）
FMT组	9.34（1.46~18.15）	3.93（0.66~12.05）	9.11（0.94~22.12）
活化T细胞（%）			
对照组	14.07（8.47~30.68）	30.07（16.52~48.89）	13.54（4.45~35.29）
FMT组	18.92（2.50~32.50）	35.58（11.88~49.02）	18.25（6.30~29.12）
静止T细胞（%）			
对照组	36.42（28.24~56.26）	20.98（9.34~43.78）	35.89（21.62~46.47）
FMT组	41.79（22.44~73.17）	22.43（10.34~40.90）	43.50（15.20~73.82）

**注** allo-HSCT：异基因造血干细胞移植；GI-aGVHD：胃肠道急性移植物抗宿主病；FMT：粪菌移植；Treg：调节性T细胞。与对照组比较，^a^*P*<0.05

五、FMT治疗对炎症因子及REG3α的影响

FMT组与对照组外周血炎症因子及REG3α浓度在GI-aGVHD发病前（基线）、发病时差异无统计学意义（*P*>0.05）。治疗后，FMT组IL-6、REG3α浓度低于对照组（*P*＝0.040、*P*＝0.021），IL-10浓度高于对照组（*P*＝0.024）（表4）。

**表4:** 两组GI-aGVHD患者治疗前后外周血炎症因子及胰岛衍生蛋白3α（REG3α）检测结果［*M*（范围）］

组别	基线	发病时	治疗后
IL-2（ng/L）			
对照组	0.55（0.21~2.63）	1.43（0.72~4.43）	0.44（0.02~1.08）
FMT组	0.60（0.01~2.22）	1.53（0.71~5.12）	0.64（0.01~2.42）
IL-4（ng/L）			
对照组	0.62（0.37~1.33）	0.02（0.01~0.12）	0.52（0.02~2.04）
FMT组	0.68（0.01~2.99）	0.01（0.01~1.30）	0.77（0.01~3.66）
IL-6（ng/L）			
对照组	4.37（3.92~9.63）	43.78（26.88~74.59）	6.82（2.40~8.91）
FMT组	3.05（1.02~10.61）	21.22（7.58~97.53）	4.15（1.91~5.71）^a^
IL-10（ng/L）			
对照组	12.95（5.90~17.12）	3.20（3.09~4.26）	7.51（4.10~9.58）
FMT组	12.81（5.90~18.78）	3.31（2.11~5.58）	12.11（5.69~20.36）^a^
TNF-α（ng/L）			
对照组	0.22（0.01~0.67）	0.77（0.39~1.78）	0.30（0.01~0.83）
FMT组	0.02（0.01~0.82）	1.08（0.07~2.80）	0.18（0.01~1.60）
IFN-γ（ng/L）			
对照组	0.11（0.01~0.46）	0.69（0.32~1.37）	0.19（0.01~0.62）
FMT组	0.02（0.01~0.67）	0.61（0.02~1.63）	0.02（0.01~1.35）
REG3α（µg/L）			
对照组	37.75（7.90~87.10）	303.90（246.90~342.10）	74.35（33.50~139.50）
FMT组	25.60（10.30~86.90）	304.80（58.40~805.20））	30.70（10.50~105.00）^a^

**注** GI-aGVHD：胃肠道急性移植物抗宿主病；FMT：粪菌移植；TNF-α：肿瘤坏死因子-α；REG3α：胰岛衍生蛋白3α；IFN-γ：γ干扰素。与对照组比较，^a^*P*<0.05

六、预后分析

FMT组、对照组移植后1年OS率高于对照组［（65.1±13.1）％对（24.0±14.5）％，*P*＝0.017］，PFS率高于对照组［（49.9±15.7）％对（22.5±14.0）％，*P*＝0.044］。

## 讨论

在allo-HSCT后，肠道微生物群结构的变化与aGVHD和预后密切相关，提示可通过调节肠道菌群干预aGVHD[12]。本研究分析了29例糖皮质激素耐药GI-aGVHD的临床资料，其中19例接受了FMT治疗，与治疗前患者的肠道菌群及未接受FMT治疗的患者相比，大多数FMT后肠道菌群的多样性及丰度较治疗前及对照组显著改善，结果与Defilipp等[5]的研究相似。此外，FMT后微生物群的组成明显恢复，在多数患者中有益菌群如拟杆菌、梭菌增加，同时肠杆菌等促炎肠道菌群减少。

研究显示，FMT治疗GI-aGVHD的总体缓解率可达74％，CR率约为50％[6]–[7],[13]。在本研究中，多数患者FMT后腹泻和腹痛症状减轻，CR率和有效（CR+PR）率分别为68.4％、78.9％，FMT组具有更高的移植后1年OS率（*P*＝0.017）和PFS率（*P*＝0.044），未观察到FMT相关感染。

肠道菌群及其代谢产物通过与免疫细胞的直接和间接作用而影响机体免疫系统，从而加重或缓解aGVHD[14]。研究显示，可产生短链脂肪酸（SCFA）的梭状芽孢杆菌通过上调Treg细胞发挥抗炎作用[15]，促进allo-HSCT后肠道上皮损伤的修复及减少细胞凋亡，从而改善aGVHD[16]。梭菌属（毛螺菌科及瘤胃球菌科）的减少以及肠杆菌的增加与Treg/Th17失衡有关，从而诱发aGVHD的发生[17]。此外，梭状芽孢杆菌Blautia丰度增加与致死性aGVHD发生率降低和OS率提高有关[18]。Zeng等[19]发现来自致病性肠杆菌科的代谢物脂多糖可促进Th17介导的炎症反应。Sofi等[20]观察到脆弱拟杆菌抑制致病性T细胞激活并阻止其向靶器官（如肝脏和肠道）迁移，同时增加时Treg细胞表达上调。本研究中，FMT治疗后CD3^+^、CD8^+^细胞下降、CD4^+^、Treg和CD4^+^/CD8^+^细胞比值升高，提示FMT可能通过减少致病性细胞毒性T细胞活化及增加产酸梭菌来上调Treg细胞表达，从而改善aGVHD。

炎症细胞因子在aGVHD的发病中起重要作用。预处理引起的肠道损伤可导致肠黏膜通透性增加，从而促进肠道细菌的位移，增强炎症细胞因子反应，为T细胞活化和启动提供了环境[21]。肠道菌群已被证实通过产生IL-10和Th2相关细胞因子以及诱导Treg细胞发挥抗炎作用。IL-10是典型的抑制aGVHD应答因子，能有效抑制分泌IL-2、IL-6、TNF-α等的Th1细胞和B细胞生成的细胞因子[22]。IL-6主要由间充质来源的细胞释放，通过多种机制促进aGVHD的发生和发展（增加Th1和Th17细胞亚群、减少Treg细胞和直接细胞毒作用等）[23]。我们的研究显示，FMT通过增加肠道菌群的多样性、减少促炎因子IL-6的分泌并促进抗炎因子IL-10分泌而改善GI-aGVHD的病程。

REG3α蛋白作为一种C型凝集素，主要由胃肠道上皮细胞产生，特别是Paneth细胞，其作为一种抗菌蛋白具有保护肠黏膜的作用。研究表明，REG3α在GI-aGVHD患者中显著升高，与GI-aGVHD的发生和严重程度密切相关，REG3α是GI-aGVHD的重要生物标志物[24]。本研究发现FMT治疗后GI-aGVHD患者外周血中REG3α较对照组显著下降，提示FMT可能通过改善内皮损伤及恢复肠道屏障及完整性来减轻GI-aGVHD。

综上，本研究结果显示，FMT治疗糖皮质激素耐药GI-aGVHD具有良好疗效，通过促进肠道菌群多样性的恢复，诱导同种异体反应由免疫、炎性失衡向再平衡的调节，从而达到治疗GI-aGVHD的目的，其免疫调控机制有待进一步深入研究。
